# Outcomes and Complications of Brainstem Arteriovenous Malformation (AVM) Embolization: A Systematic Review

**DOI:** 10.7759/cureus.94749

**Published:** 2025-10-16

**Authors:** Ali K. Al-Shalchy, Nooruldeen H. Ali Al-Khafaji, Abdullah K. Alqaraghuli, Mohammed Bani Saad

**Affiliations:** 1 Department of Surgery, College of Medicine, University of Baghdad, Baghdad, IRQ; 2 Department of Neurology, Medstar Washington Hospital Center, Washington, USA; 3 Department of Surgery, Al-Kindy Teaching Hospital, Baghdad, IRQ

**Keywords:** arteriovenous malformation, brainstem, complications, embolization, treatment outcomes

## Abstract

Brainstem arteriovenous malformations (AVMs) are rare, high-risk lesions with controversial endovascular management. We systematically reviewed the literature to clarify obliteration rates, complications, and mortality related to embolization of intrinsic brainstem AVMs and to situate the technique within modern multimodal care. Following Preferred Reporting Items for Systematic Reviews and Meta-Analyses (PRISMA) 2020 guidance, PubMed and Scopus were searched without date limits using combined terms related to brainstem structures (midbrain, pons, medulla oblongata), arteriovenous malformations, and embolization. Original English-language reports that provided detailed clinical or radiographic outcomes after endovascular treatment were eligible. Two reviewers independently screened records, extracted data, and assessed quality with ROBINS-I or CARE. Due to heterogeneity, results were summarized descriptively.

Nine studies (aged 13-79 years) met the criteria. Hemorrhage was the initial presenting event in 62%, followed by cranial nerve or long-tract deficits. Embolic agents used included n-butyl-cyanoacrylate, Onyx, glue, and coils; most lesions were treated in one (60%) or two sessions. Reported nidus obliteration ranged from 23% to 100%; complete angiographic cure was achieved in all patients in three small series that employed either the Pressure-Cooker Technique or adjunctive microsurgery/radiosurgery. Procedure-related morbidity included new neurological deficits (up to 24%), infarction (7%), and rebleed (10%). The 30-day mortality ranged from 0% to 5% across series. Median follow-up was 12 months (range 1-48), with a few late hemorrhages. Endovascular embolization can achieve meaningful flow reduction and, in selected cases, complete obliteration of brainstem AVMs, but success and safety vary widely.

## Introduction and background

Brainstem arteriovenous malformations (AVMs) are rare and complex vascular lesions, accounting for only 2-6% of all intracranial AVMs [1]. Their deep-seated location in the brainstem, an eloquent area of the brain responsible for maintaining vital neurological functions, presents significant challenges in the diagnosis and treatment of this disorder. Due to their proximity to vital structures, these lesions are frequently associated with high morbidity and mortality [1]. The main clinical manifestation of brainstem AVMs is hemorrhage, which may occur in up to 92% of cases. The annual hemorrhage rate for untreated lesions can be as high as 15-17.5%, with devastating consequences including severe neurological deficits or death [2,3]. Brainstem AVMs compared to their supratentorial counterparts display more aggressive behavior with higher rates of initial hemorrhage and poorer outcomes after rupture [2,3]. Management of brainstem AVMs is based on a multimodal approach using microsurgery, endovascular embolization, and stereotactic radiosurgery [3,4]. Complete nidus obliteration remains difficult due to the anatomical and functional limitations of the brainstem [4].

Residual or partially treated AVMs carry an increased risk of rupture, thus demanding precise treatment planning and careful long-term follow-up [4]. Among treatment modalities, radiosurgery and microsurgery have demonstrated the highest rates of nidus obliteration, though at the expense of potential neurological deterioration in complex cases [1]. Recent advances in surgical techniques and imaging, along with improvements in interventional equipment, have greatly expanded treatment possibilities for brainstem AVMs. Despite advances in endovascular techniques, the literature on embolization outcomes in brainstem AVMs remains fragmented, often limited to isolated case reports or small series without pooled analysis. There is no comprehensive synthesis focusing exclusively on the safety profile, occlusion rates, and post-procedural complications of embolization in these anatomically critical lesions. Addressing this gap, the present systematic review aims to consolidate available evidence to provide a clearer understanding of embolization efficacy and risk in brainstem AVMs.

## Review

Methods

Study Design

**Figure 1:**
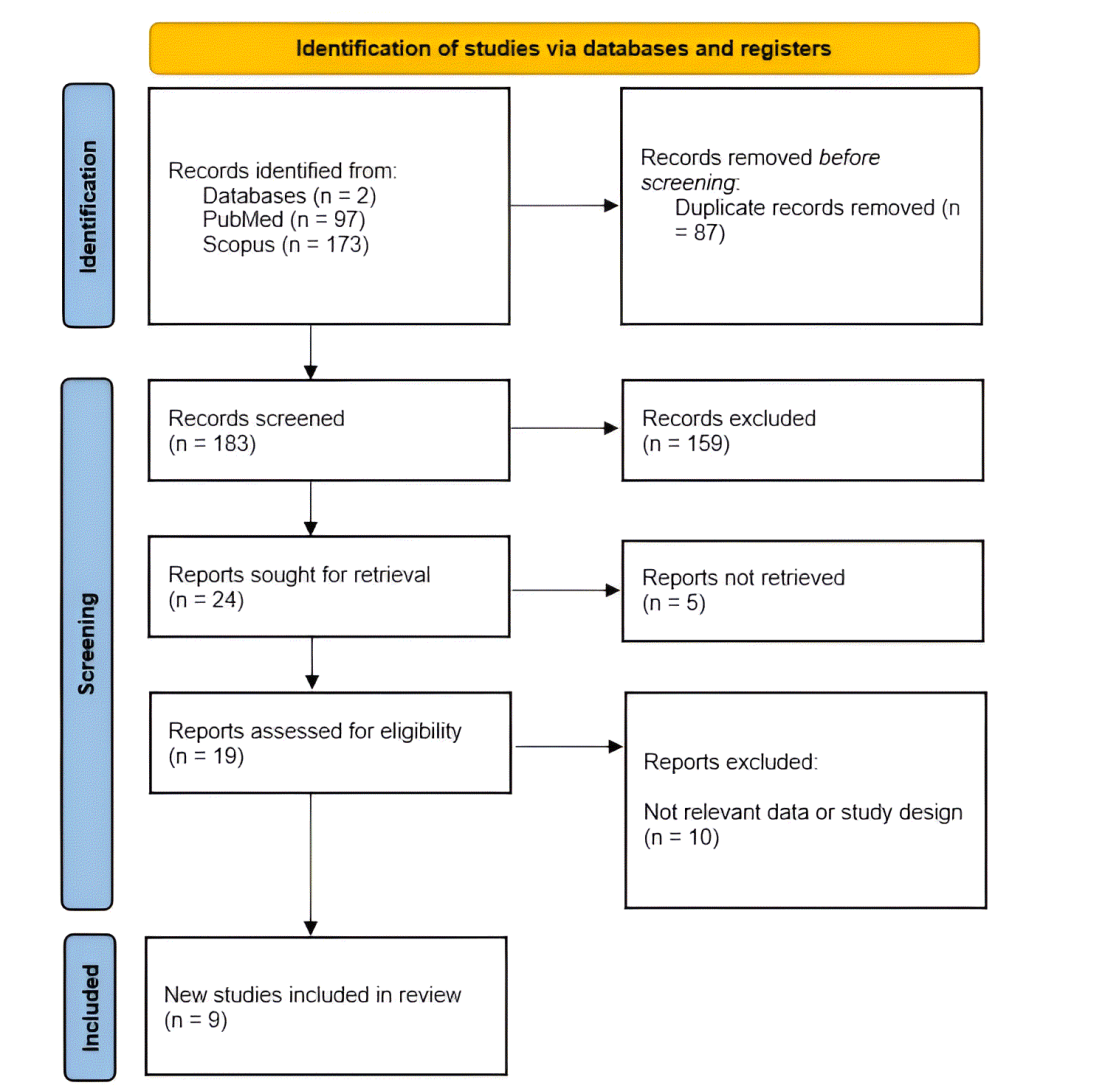
PRISMA flowchart of the included studies PRISMA: Preferred Reporting Items for Systematic Reviews and Meta-Analyses

This review was conducted following the Preferred Reporting Items for Systematic Reviews and Meta-Analyses (PRISMA) guidelines 2020 to ensure methodological rigor and transparency (Figure 1) [5]. A comprehensive literature search was performed in PubMed and Scopus databases to identify relevant studies on brainstem AVMs treated with embolization. The search terms were ("brainstem" OR "midbrain" OR "pons" OR "medulla oblongata") AND ("AVM" OR "arteriovenous malformation") AND ("embolization" OR "endovascular treatment" OR "endovascular embolization")

Study Selection

All identified articles were imported into Rayyan, a systematic review management tool that facilitates efficiency in screening and collaboration among reviewers. Titles and abstracts were screened for relevance independently by two reviewers. Full-text articles of the potentially eligible studies were retrieved to assess their eligibility for inclusion in the review using predefined inclusion and exclusion criteria. Any discrepancies were resolved through discussion, with recourse to a third reviewer when required. The inclusion criteria for this systematic review encompassed studies that reported outcomes and complications of brainstem AVM embolization. Eligible studies included original research articles, such as randomized controlled trials, cohort studies, case-control studies, and case reports, regardless of publication date. Only studies providing quantitative or qualitative data on embolization techniques, clinical outcomes, or complications were considered. Besides this, the studies had to be published in English. Review articles, editorials, and abstracts of conferences were also excluded. Moreover, studies focused on AVM outside the brainstem were excluded; furthermore, articles with no specific data about the outcome after embolization were excluded from the review process.

Data Extraction

Extracted variables included study characteristics such as authors, year, study design, sample size, and study location. Patient demographics, including age, sex, and presenting symptoms, were documented. Detailed AVM characteristics, such as size, location (midbrain, pons, or medulla), and Spetzler-Martin Grade, were also collected. Treatment details were recorded, including embolization materials and techniques, as well as any combination therapies (e.g., surgery, stereotactic radiosurgery). Outcomes of interest included obliteration rates, functional status, and complication rates, including neurological deficits and hemorrhage. Follow-up data encompassed the duration of monitoring and the recurrence of hemorrhage or complications.

Quality Assessment

The methodological quality and risk of bias of included studies were independently assessed using the Risk of Bias in Non-Randomized Studies of Interventions (ROBINS-I) tool [6] and CARE guidelines [7] to evaluate adherence to standardized reporting practices.

Data Synthesis

Descriptive statistics summarized study characteristics, patient demographics, and treatment outcomes. Outcomes stratified by treatment modality and AVM location were analyzed for subgroup analysis. Due to heterogeneity in study designs, patient populations, and reported outcomes, meta-analyses could not be conducted.

Results

Patient Demographics and Study Characteristics

This review included nine studies, a mix of case reports and retrospective case series. The number of patients analyzed across these studies varied, with a total of 33 patients. The mean age of patients ranged from 13 to 64 years, with the majority falling within the third to fifth decades of life. Hemorrhagic events, including subarachnoid and intracranial hemorrhages, were predominant, with 12 hemorrhages reported in one retrospective case series alone. The nonbleeding symptoms included headache, vertigo, right-sided trigeminal neuralgia, and neurological deficits such as unsteady gait, dysarthria, and diplopia. Follow-up periods ranged from one month to 48 months (Table 1).

**Table 1:** Summary of included studies characteristics

Study ID	Authors	Year	Study design	n	Age (Mean ± SD)	Sex (M/F)	Presenting symptom	Follow-up Period in months (Mean ± SD)
1	Liu et al. [8]	2003	Case Series	6	32.5 ± 11.4	3M/3F	One case of subarachnoid hemorrhage, one case of headache accompanied by unsteady gait, one case of pontine hemorrhage, and two cases of midbrain hemorrhage. Additionally, one patient presented with hydrocephalus.	28.4 ± 17.66
2	Morihiro et al. [9]	2010	Case Report	1	64 ± 0	Male	Headache	1 ± 0
3	Jin et al. [10]	2017	Retrospective Case Series	13	29.9 ± 12.25	8M/5F	12 Hemorrhage 1 Headache	45.2 ± N/A
4	Lim et al. [11]	2019	Case Report	1	29 ± 0	Male	Sudden onset of severe bilateral headache, blurred vision, and numbness on the right side of his face and tongue.	3 ± 0
5	Rao and Giron [12]	2020	Case Report	1	32 ± 0	Male	Dizziness, gradually progressive vertigo for two years, with unstable gait, dysarthria, and occasional diplopia.	12 ± 0
6	Cortese et al. [13]	2021	Retrospective Case Series	8	48 ± 12.8		Intracranial hemorrhage	25 ± 28.24
7	Das et al. [14]	2023	Case Report	1	50 ± 0	Female	Right‑sided trigeminal neuralgic pain	48 ± 0
8	Hirata et al. [15]	2023	Case Report	1	13 ± 0	Female	Unconsciousness and anisocoria	6 ± 0
9	Li and Yu [16]	2024	Case Report	1	43 ± 0	Male	Headache	3 ± 0

The quality assessment of the included studies revealed variability in methodological rigor. While most studies demonstrated adequate reporting of patient characteristics and interventions, some exhibited moderate to serious risk of bias due to incomplete follow-up data and lack of standardized outcome measures (Tables 2, 3).

**Table 2:** Quality assessment of case reports using CARE guidelines CARE : Consensus-based Clinical Case Reporting

Author, Year	Patient Information	Clinical Findings	Diagnostic Assessment	Therapeutic Interventions	Follow-up Outcomes	Discussion/Conclusions	Overall Quality
Morihiro et al., 2010 [9]	Comprehensive	Detailed	Thorough	Well-documented	Reported	Relevant	High
Lim et al., 2019 [11]	Comprehensive	Detailed	Thorough	Well-documented	Reported	Relevant	High
Rao and Giron, 2020 [12]	Comprehensive	Detailed	Thorough	Not Well-documented	Reported	Relevant	Moderate
Das et al., 2023 [14]	Comprehensive	Detailed	Thorough	Well-documented	Reported	Relevant	High
Hirata et al., 2023 [15]	Comprehensive	Detailed	Thorough	Well-documented	Reported	Relevant	High
Li and Yu, 2024 [16]	Comprehensive	Detailed	Thorough	Well-documented	Reported	Relevant	High

**Table 3:** ROBINS-I assessment of the included studies ROBINS-I: Risk Of Bias In Non-randomised Studies–of Interventions

Author, Year	Confounding	Selection of Patients	Classification of Interventions	Deviations from Intended Interventions	Missing Data	Measurement of Outcomes	Selection of Reported Results
Liu et al., 2003 [8]	Moderate	Moderate	Low	Moderate	Low	Moderate	Moderate
Jin et al., 2017 [10]	Moderate	Low	Moderate	High	Moderate	Moderate	Moderate
Cortese et al., 2021 [13]	Moderate	Moderate	Low	Moderate	Low	Low	Low

Treatment Modalities and Outcomes

The various embolic agents used for the embolization procedure included n-butyl cyanoacrylate, Onyx, glue, and coils. The number of sessions for embolization per patient varied from 1 to 2 sessions (Table 4). Several studies also incorporated adjuvant therapies, such as open surgery and Gamma Knife radiosurgery (GKS), to achieve further obliteration or reduce flow in residual AVMs. The rates of obliteration achieved through endovascular embolization varied significantly. In some cases, such as Liu et al. [8], obliteration ranged from 50% to 100%, depending on the number of embolization sessions. Complete obliteration was achieved in several instances, such as in Lim et al. (100%) [11], while others reported partial obliteration, such as Rao and Giron [12], where only effective flow reduction was achieved. Li and Yu also reported complete obliteration (100%) [16]. Notably, several cases utilized specific techniques to enhance outcomes. For example, Li and Yu [16] reported employing the Pressure Cooker Technique with Onyx, achieving 100% obliteration. Similarly, Hirata et al. [15] also reported the use of endovascular as well as surgical approaches to achieve near-complete obliteration and thus demonstrated the benefits of using multimodal management in selected cases.

**Table 4:** Treatment modalities and outcomes for brainstem AVMs AVM: Arteriovenous Malformation

Study ID	Authors	AVM Location (Midbrain/Pons/Medulla)	AVM Size (cm)	Spetzler-Martin Grade	Primary Treatment	Adjuvant Treatment	Embolization Agent Used (Glue/Onyx/Coils)	No. of Embolization Sessions	% Obliteration Achieved
1	Liu et al. [8]	Midbrain (2), Pons (2), Pontomedullary (2)	mean 4.44 ± 2.94 cm³	-	Endovascular embolization	-	n-butyl cyanoacrylate (NBCA)	3 cases: 1 session, 3 cases: 2 sessions	50–100%
2	Morihiro et al. [9]	Midbrain	-	III	Endovascular embolization	-	n-butyl cyanoacrylate (NBCA)	1	Nearly 100%
3	Jin et al. [10]	Midbrain (10), Pons (2), Pontomedullary (1)	1–7	-	Endovascular embolization	Radiosurgery (Gamma Knife)	Onyx	1-2	23% fully occluded, remainder partial
4	Lim et al. [11]	Midbrain	-	-	Endovascular (transarterial and transvenous embolization)	-	Onyx	2	100%
5	Rao and Giron [12]	Midbrain, Pons, Cerebellum	2.8 × 3.4 × 4.2	IV	Conservative	Endovascular	Onyx	1	Partial
6	Cortese et al. [13]	Posterior midbrain (tectum mesencephali)	1.13 ± 0.33	III	Endovascular embolization	-	Glue (6), Onyx (2)	1	63%
7	Das et al. [14]	Trigeminal root entry zone, intrinsic pontine artery	-	-	Endovascular embolization	Surgery	Coils and Onyx	1	Partial (residual treated with GKS)
8	Hirata et al. [15]	Midbrain (thalamoperforating artery)	-	III	Endovascular (coil and glue embolization)	Gamma Knife Radiosurgery	Glue and Coils	1	Partial (feeder occlusion)
9	Li and Yu [16]	AICA, Brainstem involvement	-	Not reported	Endovascular (Pressure Cooker Technique, Onyx)	Ommaya catheter with a reservoir was inserted into the frontal horn of the lateral ventricle to aspirate cerebrospinal fluid	Onyx (Pressure Cooker Technique)	1	100%

**Figure 2:**
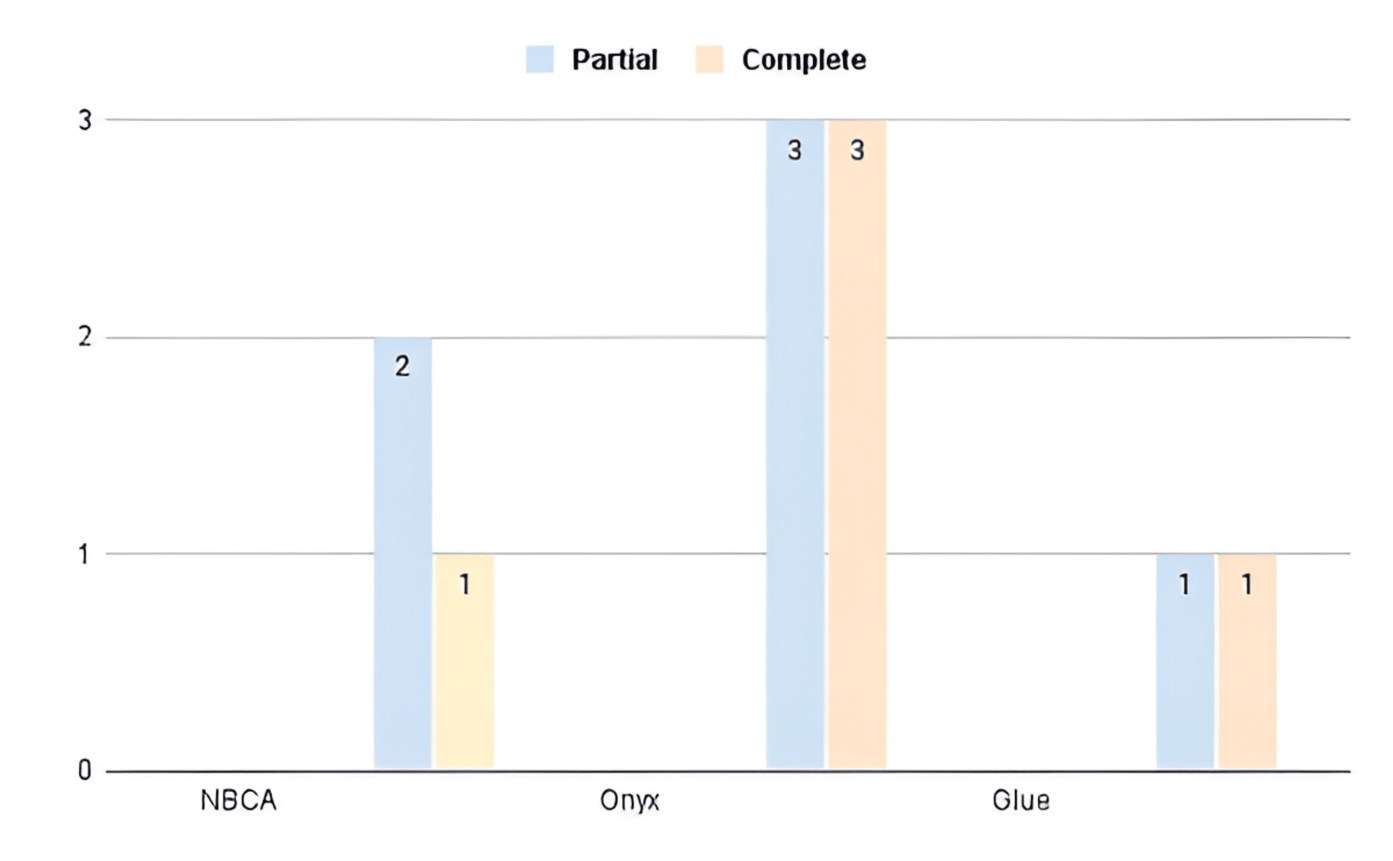
Distribution of partial and complete obliteration outcomes based on embolic agents This figure includes only the 11 patients for whom both the embolization agent (NBCA, Onyx, or glue) and obliteration outcome (partial or complete) were explicitly reported. Cases with missing or qualitative-only outcome data were excluded to ensure consistency in visualization.

Not all the uses of various treatments uniformly resulted in total obliteration, as some had partial obliterations or needed additional interventions afterward (Figure 2). For instance, Jin et al. [10] reported only 23% full obliteration, with residual nidus requiring close follow-up or additional treatment. Similarly, Cortese et al. [13] achieved 63% obliteration, with further reduction necessary via adjuvant therapy.

Complications, Risk Factors, and Mortality

Hemorrhagic events were a common presentation, with several studies reporting cases of intracranial hemorrhage, subarachnoid hemorrhage, or localized brainstem infarctions post-treatment. For example, Jin et al. [10] reported 12 patients with intracranial hemorrhages and five patients with brainstem infarctions, underscoring the high baseline risk of these lesions (Table 5). Post-embolization morbidity was reported in several series, mainly as transient or permanent neurological deficits attributable to ischemia, infarction, or cranial nerve involvement. Across the included studies, procedure-related morbidity ranged from 10% to 24%, primarily involving hemiparesis, ataxia, oculomotor or facial palsy, and dysarthria. Most deficits were transient and improved during follow-up, yet permanent deficits were occasionally observed when embolization was performed near critical perforating arteries or deep brainstem nuclei. These findings underscore the high procedural sensitivity of embolization in eloquent territories, even when mortality rates remain low or absent.

**Table 5:** Complications, risk factors, and mortality associated with brainstem AVM treatments AVM: Arteriovenous Malformation

Study ID	Authors	Hemorrhage	Hydrocephalus	Ischemia	Neurological Deficit	Infection	Anesthesia-related	Timing (Intra-Op/Immediate Post-Op/Delayed)	Management Strategy	Resolution (Full/Partial/-)	Proposed Risk Factors	Mortality Rate (%)
1	Liu et al. [8]	-	1 patient	Acute ischaemia in the embolised region in 1 patient	Mild deficit in 1 patient (diplopia)	-	-	Immediate Post-Op	Endovascular management of the AVM in the lower dorsal thalamus and upper midbrain was attempted before shunting, as the latter might have been unnecessary after the AVM-related pressure had been relieved. The patient was heparinised and his INR was maintained at levels 2.5–3 times the control.	Full	-	0
2	Morihiro et al. [9]	Subarachnoid hemorrhage	Yes	-	Transient abducens nerve palsy Truncal Ataxia	-	-	Hydrocephalus was pre-op. Abducent nerve palsy post-op. Ataxia was Delayed (1 month post-embolization)	ventriculostomy, Conservative.	Partial	-	0
3	Jin et al. [10]	Intracranial hemorrhage in 12 patients	-	Brainstem infarcts in 5 patients	Hemiplegia, ataxia, oculomotor paralysis (in 4 cases)	-	-	ICH pre-op. Others, Immediate Post-Op	Onyx embolization + Gamma Knife Radiosurgery	Partial in the majority, 2 deaths	Excessive reflux of Onyx, microcatheter entrapment	15.4
4	Lim et al. [11]	Fourth ventricle hemorrhage	-	-	Dysarthria, discoordination, diplopia	-	Shortly after completion of the procedure, the patient experienced tachycardia to 120 beats/min with anMAP of 103 mmHg.	Post-Op	A 12-mg dose of adenosine was administered, improving the heart rate to approximately 100 beats/min with a steady MAP of 73 mmHg.	Partial; improved symptoms over time	High flow AVM; technical difficulty in transvenous embolization	0
5	Rao and Giron [12]	-	-	-	Persistent vertigo, ataxia, dysarthria	-	-	Immediate Post-Op	Treatment of Major depressive disorder	Partial; AVM inoperable	Large AVM, complex anatomy, multiple intranidal aneurysms	0
6	Cortese et al. [13]	One postprocedural hemorrhage	-	One cerebellar ischemic event without clinical consequence	-	-	-	Immediate Post-Op	-	64% overall obliteration	Multiple feeders; nidus complexity	0
7	Das et al. [14]	-	-	-	-	-	-	-	-	Full	Complex anatomy, flow-related feeding artery aneurysm	0
8	Hirata et al. [15]	-	-	-	Mild residual oculomotor palsy, hemiparesis	-	-	After 6 months	Conservative	Full	Fragility of thalamoperforating artery aneurysm	0
9	Li and Yu [16]	-	-	-	Transient facial paralysis, hearing impairment	-	-	Immediate Post-Op	Rehabilitation	Full	Fragility of AICA, small feeding artery, nidus aneurysm	0

Neurological deficits were also one of the significant post-treatment outcomes. Some of them presented mild transient deficits such as diplopia and sensorineural hearing loss, whereas severe deficits presented hemiplegia, ataxia, and oculomotor paralysis. In the report by Lim et al., post-operative complications included dysarthria and discoordination [11]. Symptoms diminished with time and showed that there was some potential for recovery even after the initial deficits. Ischemic events were rare but remarkable. In the study by Cortese et al., one case that had cerebellar ischemia without clinical consequences was reported [13]. However, Jin et al. reported excessive reflux of Onyx, a complication that underscores the technical challenges of embolization procedures in highly eloquent regions [10]. Various proposed risk factors influencing complications and outcomes included the nidus' proximity to critical brainstem structures, high-flow dynamics, multiple feeding arteries, and the fragility of surrounding vasculature. Complex AVM anatomy with larger sizes and numerous feeders was associated with both increased risks of procedural complications and incomplete obliteration, as observed by Rao and Giron [12]. Indeed, in most of the reviewed studies, mortality rates were generally low. 

Discussion

Brainstem AVMs represent a significant clinical challenge due to their location in eloquent areas of the brainstem, posing risks of severe complications and treatment-associated morbidity [1-3]. This review analyzed the outcomes and complications of 9 series with 33 patients for evaluating the effectiveness and safety of endovascular embolization as either primary or adjuvant therapy in brainstem AVMs. The heterogeneous spectrum of the series included the highest number of cases presenting as a hemorrhagic event. Jin et al. reported 12 cases of hemorrhages [10]. Non-hemorrhagic symptoms, such as headaches, vertigo, and neurological deficits (e.g., diplopia and ataxia), were also prevalent. The demographic data revealed a wide age range (13-64 years). Endovascular embolization was the primary treatment in most studies, with agents such as Onyx, NBCA, and glue being commonly used. Complete nidus obliteration rates varied across studies. Liu et al. [8] reported 50-100% obliteration rates, while Jin et al. [10] achieved complete obliteration in only 23% of cases, indicating the technical challenges of treating brainstem AVMs. Advanced techniques, such as the Pressure Cooker Technique described by Li et al. [16], showed 100% obliteration, thus indicating that with appropriate tailored approaches, better results could be achieved in selected cases. GKS and surgery were used as adjunctive treatments in some cases to treat residual or complex lesions. Hirata et al. used endovascular embolization combined with GKS or open surgery and achieved near-complete obliteration [15]. However, despite multimodal approaches, partial obliteration remained common, as observed in Cortese et al. [13] and Rao and Giron [12], underscoring the need for further refinement of treatment strategies. Treatment-associated complications were significant and varied widely across studies. Jin et al. reported intracranial hemorrhages in 12 patients [10]. Neurological deficits, such as hemiplegia, ataxia, and oculomotor paralysis, were common, particularly in cases with incomplete obliteration or recurrent hemorrhage. These complications underline the delicate balance of managing such lesions, considering the proximity to critical structures of the brainstem. Despite these risks, the overall mortality rate was low, with only Jin et al. reporting a mortality rate of 15.4% in their cohort, primarily due to excessive hemorrhage and treatment failure [10]. Nevertheless, this work has the largest number of participants.

Compared with stereotactic radiosurgery (SRS), which achieves a long-term obliteration rate of 42-71% and favorable outcomes in about 31-66% of patients after several years of latency, embolization provides immediate hemodynamic control and is often employed as a bridging or adjunctive therapy [17]. Microsurgical resection, on the other hand, offers the highest obliteration rates (~90%) but carries significant morbidity (≈14%) and mortality (≈7%) even in specialized centers [2]. Given the eloquent nature of the brainstem, embolization’s minimally invasive nature and capacity for precise hemodynamic modulation make it particularly valuable in cases where surgery or high-dose SRS carries a prohibitive neurological risk.

It should be noted that not all included studies provided detailed obliteration data. This lack of uniform reporting limited the ability to compare results across studies and may have contributed to the wide variability observed in obliteration rates. Consequently, our findings should be interpreted with caution, as the absence of quantitative data from some series may underestimate or overestimate the true efficacy of embolization.

The heterogeneity in study designs, patient populations, and treatment modalities limits the generalizability of these findings. Case reports and small retrospective series dominate the literature, introducing potential bias and limiting statistical power. Future studies should aim for larger multicenter cohorts with standardized reporting of outcomes to better define the efficacy and safety of embolization for brainstem AVMs. Embolization of the brainstem AVM has been the mainstay treatment option with an encouraging prognosis for well-chosen patients. The complication rates remain high and obliteration varies significantly; hence, this stresses the need for an individualized treatment strategy, as well as thorough planning for embolization procedures. The techniques for embolization need to be refined and supplemented with studies dealing with optimal brainstem AVM management to develop outcomes for the patient.

Limitations

This review is limited by the predominance of case reports and small series, introducing potential publication bias and restricting generalizability. Heterogeneity in reporting embolization techniques, outcome measures, and follow-up duration hindered quantitative synthesis. Additionally, incomplete data on obliteration rates and AVM characteristics in some cases limit the robustness of pooled conclusions.

## Conclusions

Endovascular embolization remains an integral part of the multimodal management of brainstem AVMs. While it can achieve meaningful symptomatic and hemodynamic improvement in selected cases, procedure-related morbidity continues to limit its curative potential. When combined with microsurgical or radiosurgical approaches, embolization may contribute to safer and more effective outcomes. Further multicenter studies with standardized reporting are required to clarify its precise role and long-term efficacy. Future studies should be designed as multicenter studies with uniform protocols to further delineate treatment methods and fill existing gaps in the evidence.
